# ACFM: adaptive channel weighted fusion algorithm for improving small object detection performance in UAV traffic

**DOI:** 10.1038/s41598-026-39789-6

**Published:** 2026-02-11

**Authors:** Shijun Liu, Honghao Zhu, Zhenguo Yuan, Xingfei Zhu, Cheng Guo

**Affiliations:** 1 School of Computer and Information Engineering, Bengbu University, Bengbu, 233000 China; 2 National University of Life and Environmental Sciences of Ukraine, Kiev, 03041 Ukraine

**Keywords:** UAV, Small object detection, Feature fusion, Multi-scale, Adaptive, Engineering, Mathematics and computing

## Abstract

In terms of small objects in drone traffic monitoring, problems like insufficient feature representation, serious background interference, and poor multi-scale adaptability are often encountered. Especially when dealing with complex traffic situations, poor context linking between objects as well as poor detection in congested regions are more noticeable. In order to solve the above problems, we put forward an adaptive channel weighted fusion module, which is ACFM. First, we build a multi-scale refinement module, which can do cross-scale feature interaction via a downsampling-upsampling path. It is combined with a residual calibration mechanism to greatly improve both the localization consistency and the detail preservation of small objects over different resolutions of feature maps. And then, a grouped sparse mask attention module is created to reduce background noise through channel grouping and sparse gating techniques in order to enhance the local saliency features of sparse small targets. Finally, we add a channel-wise adaptive weighting by using the global context. Using an α weight generator which can change the contribution of features based on scene complexity, it get rid of traditional fixed combination plans. From the experimental results, we can see that adopting GFL as the detector, ACFM achieves better performance improvements on the widely used VisDrone2019, UAVDT dataset, and the maximum gain in mAP is more than 0.8% and 1.3% higher than the comparison methods. On the AU - AIR dataset, ACFM is still a little bit better than the other 0. 5% mAP, it is still robust in the complicated situation.

## Introduction

Contrary to general object detection problems, there are some practical problems for UAV based object detection. The objects captured by the aerial platform usually have large scale difference because of the different flight height, and many targets occupy a small area in the picture. The aerial viewpoint has more scenes and targets occlusion. And it is also complicated by like motion blur from platform being a little bit off and different lights. Consequently, the UAV detection model needs to effectively change scale and have good robustness for the difficult visual condition. Traditional Convolutional neural network (CNN)usually pays more attention on abstract semantic in deeper part which often ignores fine-grained spatial details from higher level and shallow stage feature of high-resolution part. It causes loss of effective feature information of small object gradually while propagating layer by layer, seriously restricts detection performance. To solve this problem, Feature Pyramid Networks (FPN)^1^ and its variants^2–5^ have reduced some difficulties with multi-scale object detection and combined the features at different levels. On this foundation, research like the enhanced feature correlation (EFC)^6^ strategy has also improved the models’ ability to sense small objects by modifying how features from neighboring layers are merged together. However, even though great progress has been made in terms of feature fusion, most existing strategies still use rather static or pre-defined ways to combine multi-scale information. In cases of complex scenarios, like drone aerial photos having complex backgrounds, different sizes of objects, and uneven light conditions, they can’t effectively differentiate small objects from background noises and can’t achieve perfect detection in places with many things. And so their adaptability and accuracy of detection continue to need improvement. A central and recurring problem is the desire for more feature representation of small objects at no great computational cost, so that the model can remain light and perform in real time.

In order to solve the aforementioned problem and the problem of the EFC^6^ strategy in feature fusion in complex scenes, we present an ACFM algorithm module. In this module it uses a more dynamic, detailed feature fusion system to make the model better at spotting small things in difficult situations. The basic idea of ACFM module is to create a dual-channel framework with the MSRB and GSA modules working together. It does adaptive weighted fusion on the output feature of both branches by establishing a Channel-level Adaptive Weight Mechanism (CAWM). ACFM has a feature that is not so common and that is it adjusts by itself how much for each of its parts of the features will contribute according to the inputs that are there right now in time rather than just being stuck in a static or fix-weight state like other ways of combining features. Main contribution of this paper are as follows:

1. A multi-scale refinement module has been built, which uses downsampling-upsampling pathway to achieve cross-scale feature interaction. Residual calibration mechanism is combined with this dual-path so as to increase localization consistency and small object detail retention at different resolutions of feature map.
2. Grouped Sparse Mask Attention Module. By using channel grouping and adaptive sparse threshold gating strategy, it does fine-grained attention modulation on feature maps. It enhances the local salient features of small objects and suppresses the background noise, thus increasing the response intensity of sparsely distributed small objects.
3. Proposed global context-based channel level dynamic weighting method. The mechanism modifies the dynamic weighting factor α and the weight generator to dynamically change the fusion ratio of the two feature branches according to the complexity of the scene, and thus, the flexible feature fusion strategy is more flexible than fixed weight allocation.

## Related works

Data-driven intelligent method is now becoming a kind of research paradigm that is significant in a number of engineering and information-processing fields, for example, industrial automation, security analysis, signal processing, and so forth^7–10^. Among which deep learning is paid much attention for its good hierarchy feature extract. It also can apply to other tasks of the wireless communication like the CSI feedback, channel estimate and channel prediction^11–13^beside CV. It means that deep neural networks can learn transferable representations between different domains according to the successful studies. From this we have the sections that follow on recent progress in computer vision.

### General object detection

Object detection, as an essential and important part of computer vision, it wants to know what kinds of things are in the picture and where they are. As for existing mainstream detection methods, they are generally classified as two-stage and one-stage detectors according to their processes. They are localised separately as being either anchor - based or non - anchor - based. Architectures like fast r-cnn^14^, faster r-cnn^15^, mask r-cnn^16^are based on a 2-step method. A first set of candidate object areas is produced and then sent on to separate network branches for the separate tasks of the final classification and bounding box refinement. This stepwise optimization design is usually more accurate in detection, but the cost of computation is relatively high. While the single-stage detectors like SSD^17^, the YOLO^18,19^series, GFL^20^, RetinaNet^21^, FCOS^22^, VFNet^23^, and FSAF^24^. At the root of it, single-stage detectors use a unified end-to-end network that is designed to spit out both classes and precise locations directly from the input image. Eliminates the intermediate step of proposing region proposals, so it does classification and localization all in one big computational step. So, they generally do faster inference and have better real-time performance. And in an anchor-based detection system, these is a set of defined prior geometric element of certain sizes and aspects ratio. It is named anchors. They are like initial spots, around which parts specifically dedicated to bounding box regression heads make tiny changes to locate objects more accurately. These anchor boxes give the network some advance knowledge as to where and what form the objects might take.

### Small object detection in UAV imagery

And as for small object detection, it is also an important problem in the field of computer vision, because small objects are naturally sparse in pixels, have weak feature characteristics, and are more easily affected by the background. Especially in wide-angle applications such as UAVs. There are so many different ways to solve this problem, some methods increase the input pixel size of the network^25–27^, and some increase it by changing the data^28,29^, or even developing various multi-scale network structures, which can also increase the high-level features by adding more low-level feature maps^30^. Subsequent attempts focused on improving the architecture of the network such as QueryDet^31^, CEASC^32^and UFPMP-Det^33^, these focused on concentrating on the working query set to improve feature processing and key region strengthening. EFC has been used as a feature fusion strategy to improve network bottleneck. Though there are these explorations that have made some breakthroughs in many ways, still there are some core issues: to achieve a very stable feature recognition capability in complex dynamic environment, to build information fusion mechanisms that have dynamic adjustment ability, to provide an accurate representation for very small and sparsely distributed small objects.

### Feature pyramid network

Multi-scale feature representation construction is an important scale strategy in computer vision. FPN^1^and its variant become the basis of modern detector. FPN creates more semantic-rich multi-level feature maps by combining deep-layer context with shallow detail through the top-down and lateral pathways. This multi-scale representation turns out to be very important for detecting small objects. In order to better performance, after this work, many innovations have been made: Bi-FPN^2^balances efficiency and performance via weighted bidirectional cross-scale connections and path optimization; NAS-FPN^3^automatically optimizes connection topology using neural architecture search; PANet^4^introduces a bottom-up additional path to improve bidirectional flow of information. Additionally, ASFF^33^also suggests adaptively fusing features on different levels along the spatial dimension and learns the best fusion weights for every spatial position. AugFPN^5^adds some parts to make FPN stronger. These are called Consistency Supervision, Residual Feature Improvement, and Soft RoI Selection. Also, works like A2-FPN^34^try to bring in attention mechanisms at the fusion nodes to direct effective feature aggregation but this can make the process more computationally intensive. These improvements show FPN going from fixed structures to more flexible, adaptive, and even automatic designs. At the heart of the matter is still getting multi-scale information together in a better way though that sometimes means weighing progress against processing demands.

### Attention mechanism networks

There are mainly 4 types of current attention mechanisms in CNNs, they are channel attention, spatial attention, channel-spatial hybrid attention, and self-attention. SENet^35^works via learning a channel-wise descriptor via a gating mechanism. This descriptor excites informative features and suppresses less useful ones, so it models inter-channel relationships. ECA-Net^36^uses a local cross-channel interaction method without dimension reduction. Adaptive one-dimensional convolution is used to capture the dependencies between the adjacent channels. FCANet^37^has a dual-path setup of local cross-channel interaction with global channel reordering, it dynamically changes the parameters into refined weights. EMA^38^put forth a new cross spatial learning strategy, they designed multiple scale parallel sub networks to create both short term and long-term dependency relations. Reconstruct partial channel dimensions into processing dimensions, increasing efficiency. CCNet^39^utilizes the spatial-channel relationship through the use of cross-attention to capture interactions at different scales and high-level semantics, improving feature interactions and information flow. Additionally, spatial-channel hybrid attention mechanisms such as CBAM^40^use convolution to combine cross group channel and spatial information and provide adaptive feature refinement. To make it better and better with channels and space module. Current designs hope to use the self-attention mechanisms of the Transformer model in CNN architecture to overcome the problems of global dependence caused by the convolutional operation. For example, BotNet^41^replaces some residual blocks in ResNet^42^with Multi-Head Self-Attention modules. The DETR series of models introduce Transformer architecture in detection. For example, with Self-Attention and Cross-Attention, they began using standard Transformers inside of object detection, and so now end-to-end detection is possible, and no NMS post-processing is necessary. Deformable Multi-Head Attention^43–45^, uses local sampling for global attention instead of global attention which makes it more sensitive to the location of objects but at the same time greatly reduces computational cost; Sparse attention mechanisms^46^greatly reduce the computational complexity of attention by using local sampling and dynamic selection. Also, multi-scale attention mechanism^47^can optimize the multi-scale attention for small object detection. The cross attention mechanism like^48^boosts the detection’s trustworthiness and accuracy by utilizing anchor box’s position prior so as to narrow down on the salient feature parts.

## Methodology

### ACFM overview

**Fig. 1:**
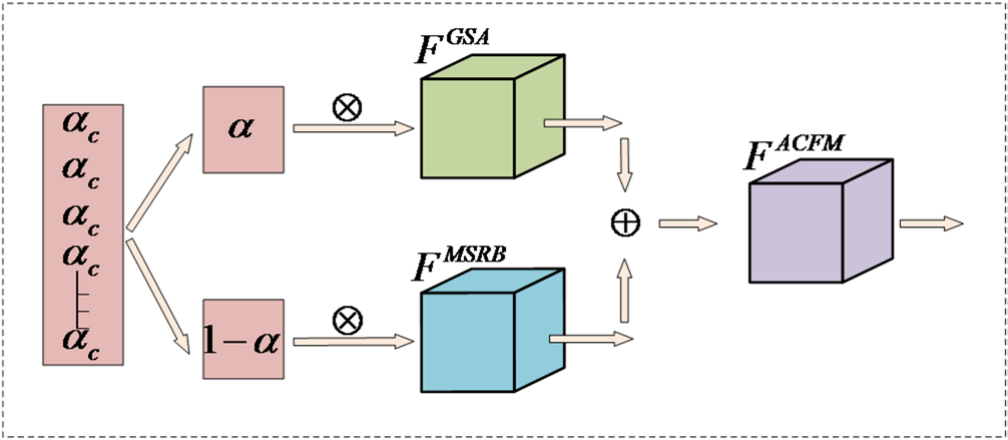
ACFM module structure diagram.

Small objects due to the limited size, features in deep network often have detail loss and lack of enough semantic information. We propose an ACFM that can improve at the same time both the local saliency and multi-scale contextual expression of small object features as shown in Fig. 1.

ACFM module is constructed with two auxiliary branch and an adaptive weighting mechanism, in GSA mechanism highlights the essential details and suppress irrelevant background with group sparse masked attention. The MSRB retains spatial information because it blends multi - scale information using down - sampling, up - sampling and path integration. Both branches output is dynamically fused using a CWAM for a balance of details and semantics. ACFM as an adaptive feature fusion module, it is plug-and-play to improve current performance in small object detection.

### Multi-scale refinement block module

**Fig. 2:**
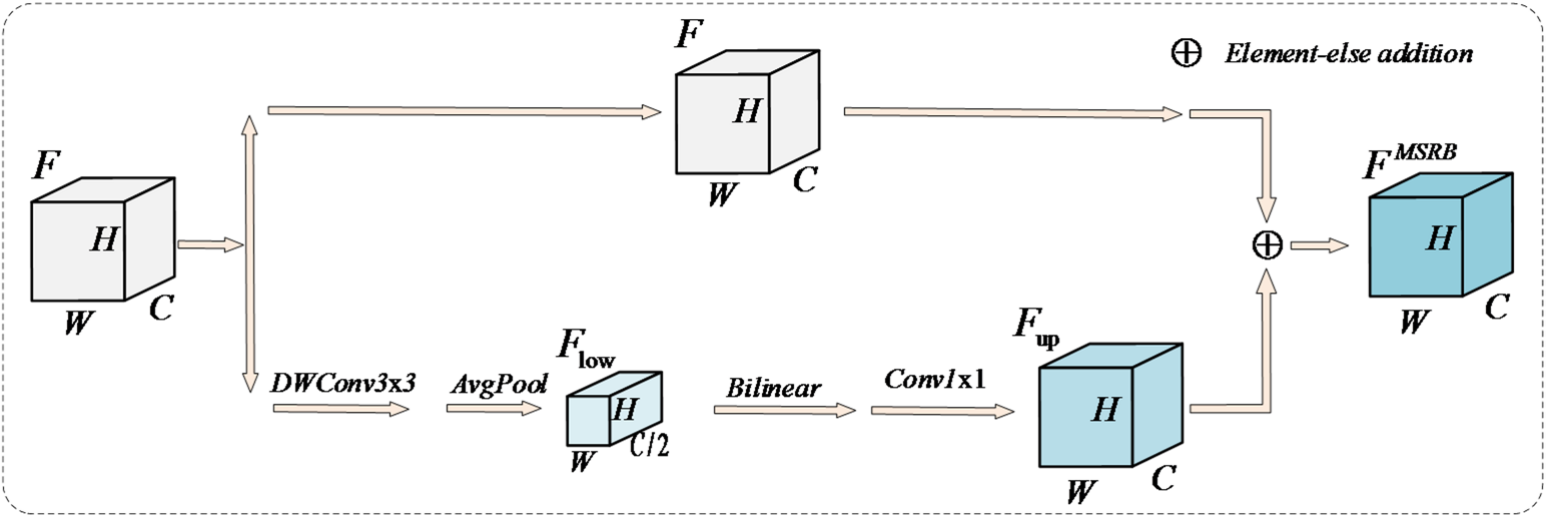
Multi-scale refinement branch MSRB structure diagram.

Drone traffic monitoring situations, object detection will have issues caused by various sizes, backgrounds and features. As can be seen from Fig. 2, the proposed solution is MSRB which is made to address these problems. This module does cross-scale feature interaction with a dual-path architecture and uses a residual calibration approach to maintain spatial detail, improving both the robustness and discriminative ability of small object features.

The first is a residual fusion strategy that keeps the original semantic and spatial information. Its advantage is to retain the spatial details of input feature directly, so that the effective information of shallow layer can be sent to the output. And this is also complementary and integrated with those features which were processed over scales, greatly improving the small object feature. So, the model keeps good vehicle object recognition even for different sizes and some background noise.

Then we go to the second path module for a feature processing workflow starting with the input features F. In order to study the detailed contextual information without the original spatial location, we use 3 × 3 depth-wise separa-ble convolution to obtain a context local context information without redundancy, as we can see in Eq. (1).

1 $${F_{dcconv}}=DWCon{v_{3 \times 3}}(F)$$

Subsequently, channel-wise dimension reduction is performed via average pooling to obtain a low-dimensional representation${F_{{\mathrm{low}}}}$, As shown in Eq. (2). This step rapidly focuses on the global distribution through dimensionality reduction, enabling the model to initially grasp the contextual relationships of small vehicle objects within large scenes and prepare for cross-scale feature fusion.

2 $${F_{low}}=AvgPool({F_{dwconv}} * F)$$

After ${F_{{\mathrm{low}}}}$ obtaining low-resolution features, To rescale it to the original feature scale, bilinear interpolation is employed for upscaling, utilizing a 1 × 1 convolution, The ${F_{{\mathrm{low}}}}$ image will be restored to its original resolution, as shown in Eq. (3).

3 $${F_{up}}={f_{1 \times 1}}(Bilinear({F_{low}}))$$

Among them${f_{1 \times 1}}( \cdot )$Indicates a 1 × 1 convolution operation, Bilinear () indicates bilinear interpolation.

Finally, the refined path outputs are element-wise summed with the identity path to yield the MSRB output, whose Eq. is given by (4).

4 $$F^{{MSRB}} = F + F_{{{\mathrm{up}}}}$$

### Group sparse mask attention module

**Fig. 3:**
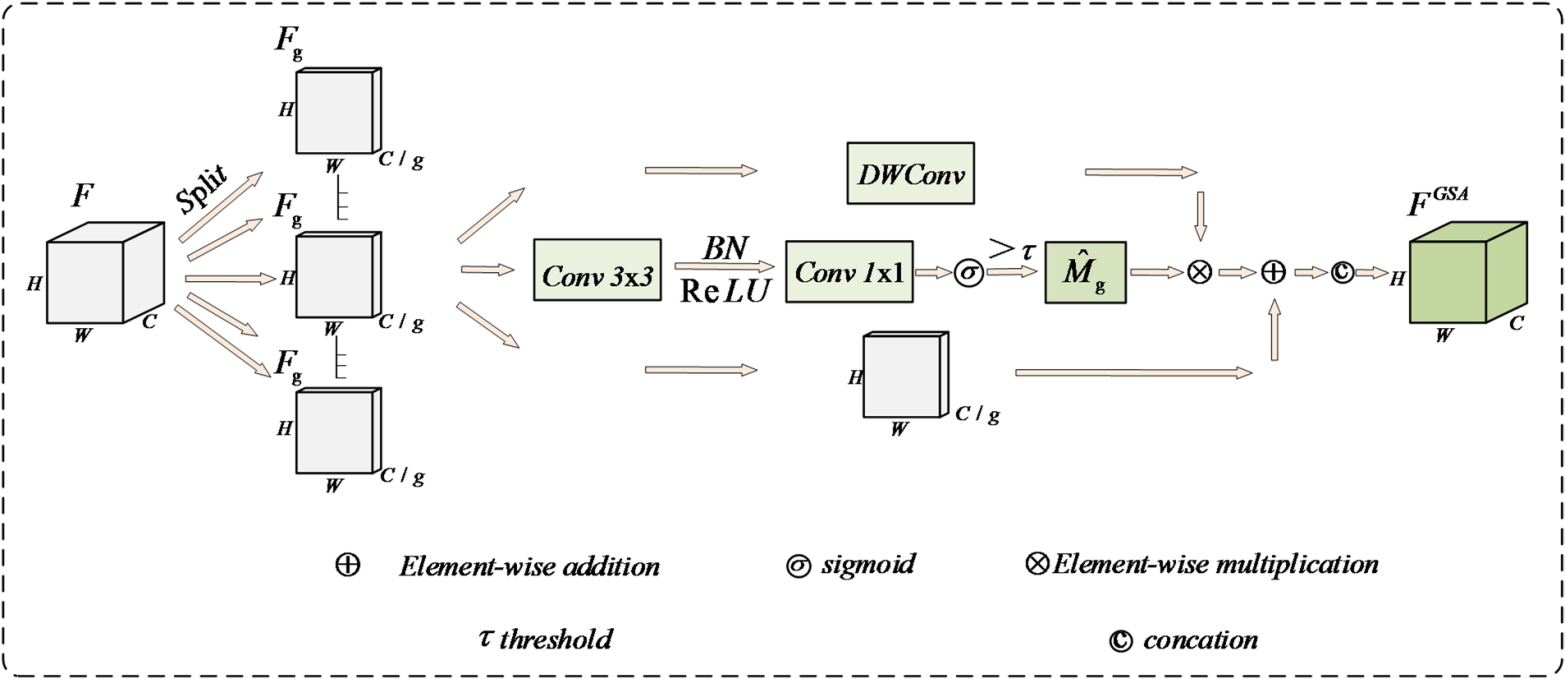
Group sparse attention branch structure diagram.

Convolutional layers often suffer from interference from background noise and redundant features in complex scenes, leading to insufficient expression of fine-grained features of objects and consequently affecting detection accuracy. To mitigate this issue, this paper proposes the GSA module. By employing channel grouping and an adaptive sparse gating mechanism, it achieves efficient enhancement of salient features and suppression of redundant ones, as show in Fig. 3.

First input the features$F \in {\mathbb{R}}{ ^{{C_{\mathrm{g}}} \times H \times W}}$Grouped into Group G by channel, each group contains $C_{{\mathrm{g}}} = C/G$ channels, yielding the grouped features, as shown in Eq. (5).

5 $$F=\{ {F^1},{F^2},...,{F^G}\} ,{F_g} \in {{\mathbb{R}}^{{C_g} \times H \times W}}$$

This operation reduces computational overhead per group and provides structural constraints for subsequent sparse modeling. Subsequently, for each feature group ${F_g}$, a 3 × 3 convolutional network is employed to sparsely activate the mask, enabling adaptive filtering of prominent detail regions. Batch normalization (BN) stabilizes the feature distribution, while the ReLU nonlinear activation function enables the network to better model nonlinear relationships and enhance responses to critical regions. Finally, the initial mask ${\hat {M}_g}$ is obtained through 1 × 1 convolutions and the sigmoid function. However, directly using masks with continuous values may result in insufficiently concentrated enhancement regions, making the model susceptible to interference from weak response areas when processing small objects. Therefore, a threshold$\tau$is introduced to binarize the values in ${\hat {M}_g}$, enabling the mask to form a spatially clearer selective distribution, as shown in Eq. (6).

6 $${\hat {M}_g}=\sigma ({f_{mask}}({F_g}))$$

Here, ${f_{mask}}( \cdot )$denotes the gate mask generation network, and $\sigma ( \cdot )$represents the sigmoid function. Subsequently, after obtaining the sparse mask, deep convolutions are employed to extract enhanced features. Then, residual fusion combines the original features with the detail-enhanced features, while the sparse mask controls the enhancement regions to effectively highlight salient details and suppress irrelevant background. Finally, the outputs from each group are reassembled into a complete feature map, yielding the GSA output, as shown in (7–8).

7 $${F_{\det ail}}={f_{dwconv}}({F_g})$$

8 $${F^{GSA}}={F_{\mathrm{g}}}+{F_{\det ail}} \odot \hat {M}$$

Among them $\odot$denotes element-wise multiplication operations. Through this design, GSA can adaptively highlight locally salient regions of small objects in complex traffic scenes, effectively suppressing noise and redundant features while maintaining controllable computational efficiency. This provides subsequent detection heads with more discriminative feature representations.

### Channel-level adaptive weights

**Fig. 4:**
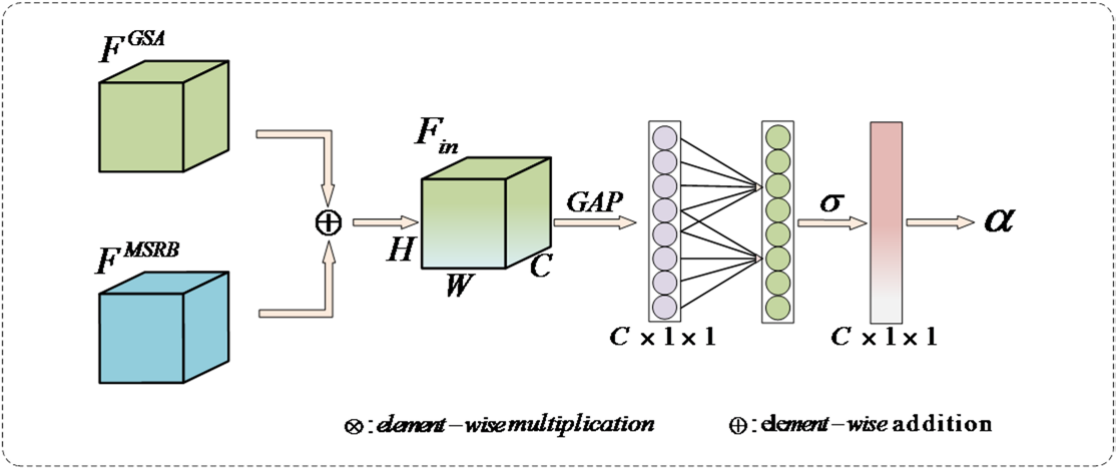
Channel-level adaptive weight structure diagram.

In small object detection tasks, traditional static weighting or fixed-ratio fusion methods often struggle to balance fine-grained features with multi-scale semantic features, particularly in complex or dense scenes. This imbalance in feature contributions can degrade detection accuracy. To address this, this paper proposes a CAWM, as illustrated in Fig. 4. By introducing a lightweight global context modeling mechanism and a channel-specific weight generation process, this module dynamically generates a weighting factor $\alpha$ to adaptively fuse the outputs from different branches.

First, the input features for CAWM are obtained by element-wise summing the output features from the GSA branch and MSRB branch to yield ${F_{in}}$:

9 $${F_{in}}={F^{GSA}} \oplus {F^{MSRB}}$$

The input feature ${F_{in}}$ then undergoes global average pooling (GAP) to compress the spatial dimension into a channel-level statistical vector *y*, as described by Eq. (10):

10 $${y_c}=\frac{1}{{H \times W}}\sum\limits_{{i=1}}^{H} {\sum\limits_{{j=1}}^{W} {{F_{in}}} } (c,i,j)$$

Subsequently, to efficiently capture local cross-channel interaction without dimensionality reduction, we employ a fast one-dimensional convolution. The kernel size *k* of this 1D convolution is adaptively determined based on the channel dimension *C*, as shown in Eq. (11):

11 $$k=\psi (C)={\left| {\frac{{{{\log }_2}(C)+b}}{\gamma }} \right|_{odd}}$$

where $|t{|_{odd}}$ indicates the nearest odd number to *t*. We set $\gamma =2$ and $b=1$ in our experiments. This adaptive kernel size allows the module to flexibly adjust the receptive field of channel interaction based on feature density. Then, the dynamic weighting factor $\alpha$ is generated by passing the convoluted features through a Sigmoid activation function $\sigma$, ensuring the weights are normalized within the range $(0,1)$, as shown in Eq. (12):

12 $$\alpha =\sigma ({\mathrm{Conv1}}{{\mathrm{D}}_k}(y))$$

Finally, the generated weight $\alpha$ is applied to adaptively fuse the features from the two branches. The GSA branch is weighted by $\alpha$, while the MSRB branch is weighted by $(1 - \alpha )$, achieving a dynamic balance as shown in Eq. (13):

13 $${F_{ACFM}}=\alpha \otimes {F^{GSA}}+(1 - \alpha ) \otimes {F^{MSRB}}$$

where $\otimes$ denotes element-wise multiplication across channels. This mechanism ensures that channels containing critical small object information are emphasized, while redundant background noise is suppressed.

## Experiments

### Experiment setup

#### Experimental implementation details

Experiments were run on MMDetection with pytorch 2.2.1 and nvidia-rtx4090gpu. On VisDrone^49^, we trained all the detectors for 15epochs with a batch size of 4 and initial learning rate 0.01. Linear warm up, 10x drop at 11th and 14th epochs. On UAVDT^50^, train detector for 6 epochs, batch size = 4, initial lr = 0.01, drop lr by 10x at 4, 5 epochs. On the AU-AIR^51^data, we also trained the model for 10 epochs with a batch size of 4, an initial learning rate of 0.01 and a 10 times reduction after the 7th and 9th epochs. In term of the experiment itself is setting the input dim for the VisDrone, UAVDT and AU-AIR dataset as 1333 * 800, 1024* 540 and 1333 * 750 respectively.

#### Datasets

VisDrone dataset is a dataset which has a lot of small objects seen by the drone. It contains 10, 209 high-resolution(2000 × 1500)aerial images of 10categories. As in previous work^6,24,25^, we employed 6, 471 images for training and 548 images for testing. UAVDT dataset, there are 23,258 training images and 15,069 testing images which have resolution about 1080*540 pixel, containing 3 kinds of vehicles (car, truck, bus). The AU - AIR dataset consists of 32,823 video frame images with a size of around 1920 * 1080 pixels, which contains 8 kinds of vehicles. We use stratified random sampling by doing a leave-one-out on the AU-AIR data set. We fixed the seed after which we took out 30% of the images for our research. The training and testing sets are manually divided in a 8:2 ratio: 7876 training images and 1970 test images.

#### Evaluation metrics

We will use some evaluation metrics to do evaluation on the model.

1. Precision (P): Proportion of samples predicted positive that are actually positive (Eq. 14). Here TP stands for true positives–those are correctly predicted as positive and really are so. FP stands for false positives–it is when something is predicted as positive even though it’s actually negative.
  14 $$P=\frac{{TP}}{{TP+FP}}$$
2. Recall (R): The percentage of samples which are actually positive samples and are predicted correctly. Its Eq. is (15). Where FN is False negatives (Predicted as negative but actual is positive).
  15 $$R=\frac{{TP}}{{TP+FN}}$$
3. Average Precision (AP): Used to evaluate single-class detection performance, its Eq. is given in (16).
  16 $$AP=\int_{0}^{1} P (r)dr$$
4. Mean Average Precision (mAP): The average of the AP values across all classes, used for comprehensive evaluation in multi-class detection tasks. N denotes the total number of classes, and $\:A{P}_{i}$represents the AP value for the i-th category, as defined by Eq. (17).
  17 $$mAP=\frac{1}{N}\sum\limits_{{i=1}}^{N} {A{P_i}}$$

### Ablation experiments results and analysis

To validate the strength and efficaciousness of the aforementioned approach concerning a situation involving UAV traffic, we have used the GFL^12^as the detector, and the EFC has been chosen as our baseline model in this research. Multiple publicly available UAV small object detection benchmark datasets were systematically experimented with, such as VisDrone2019^42^, UAVDT^43^, and AU-AIR^44^. These datasets have different traffic environment datasets and different scene characteristics. So, they can be tested well under different circumstances.

#### Analysis of MSRB

To improve the model’s ability to capture multi-scale features of small objects, we propose MSRB. Compared with traditional convolution operations, the MSRB can realize cross-scale feature interaction via a dual-path framework and keep spatial details through a residual calibration procedure. This makes the small objects in the complex traffic scene more accurate and resistant to detection, as shown in the experimental results of Table 1.

**Table 1:** Experimental results of MSRB on VisDrone, UAVDT, and AU-AIR datasets (%).

Method	VisDrone			UAVDT			AU-AIR		
	mAP↑	AP50↑	AP75↑	mAP↑	AP50↑	AP75↑	mAP↑	AP50↑	AP75↑
Baseline	28.4	48.1	29.5	14.9	25.7	15.2	15.1	36.9	9.9
+MSRB	29.0	48.7	30.0	16.0	27.8	16.9	15.6	37.5	10.3

Experimental results show that after adding MSRB, on the VisDrone dataset the mAP increases from 28.4% to 29.0%, AP50 increased 0.6%, AP75 increased 0.5%. Validates it can improve small object localization consistency and keep details. In terms of the more difficult and numerous UAVDT dataset, the mAP rises from 14.9% to 16.0% (with a relative improvement of 1.1%), and AP50 and AP75 rise by 2.1% and 1.7% respectively, showing the robustness of MSRB in multi-scale and noisy conditions. In the weak feature case of low-contrast and sparse small objects on AU-AIR dataset, mAP is still able to get 0.5% improvement, and AP50 and AP75 can get 0.6% and 0.4% improvement respectively, which shows it works well on weak feature conditions. In general, MSRB can provide good stable performance increase on all 3 datasets, it can also improve the representation of small object details, suppress the loss of features and improve the robustness of detection.

#### Analysis of GSA

To improve the discriminative ability of small object feature, this paper presents a GSA module. Unlike traditional convolution operations which can be easily disrupted by background noise and unnecessary feature interferences, GSA performs its computations using a group and sparse gating operation that is capable of intelligently suppressing the non-important responses and making the important responses stand out more with regards to the finer grained objects. In complex backgrounds, the design greatly improves the small objects’ details, which is very helpful for improving the robustness of detection. as shown in the experimental results of Table 2.

**Table 2:** Experimental results of GSA on VisDrone, UAVDT, and AU-AIR datasets (%).

Method	VisDrone			UAVDT			AU-AIR		
	mAP↑	AP50↑	AP75↑	mAP↑	AP50↑	AP75↑	mAP↑	AP50↑	AP75↑
Baseline	28.4	48.1	29.5	14.9	25.7	15.2	15.1	36.9	9.9
+GSA	28.8	48.9	30.1	15.8	27.5	16.5	15.5	37.8	10.4

From the experimental results we can see that on the VisDrone dataset when using GSA mAP goes up from 28.4% to 28.8% and AP50 & AP75 increase by 0.8% & 0.6% respectively. This argue the module’s enhanced capableness‌ to capture multi-scale object details. On UAVDT, GSA made even bigger changes: +0. 9% on mAP, but + 1. 8% and + 1. 3% on AP50 and AP75. Which means it’s quite good at discriminating occluded and crowded small things in traffic scenes. On AU - AIR dataset, mAP improves 0.4%, and AP50, AP75 improve 0.9% and 0.5% respectively. This also proves that the module is stable in complex background environment and prove the effectiveness of GSA. The results have fully demonstrated that compared with our proposal which has an advantage of our grouped sparse gating structure that is able to express more fine-grained features and suppress background noises, the performances of the detections are all improved.

#### Analysis of CAWM

To improve the discriminative representation of small-object features, we design a channel-level adaptive weighting module. Conventional convolution operations apply identical processing across all channels and therefore struggle to adapt to variations in scene complexity. In contrast, CAWM introduces dynamic weighting factor $\alpha$, which are adaptively generated according to the input feature characteristics. Channels that carry more informative cues for small objects are emphasized, while those dominated by redundant or noisy responses are suppressed. This design aims to alleviate feature inconsistency issues that commonly occur during the direct fusion of heterogeneous branches.

**Table 3:** Experimental results of CAWM on VisDrone, UAVDT, and AU-AIR datasets (%).

Method	VisDrone			UAVDT			AU-AIR		
	mAP↑	AP50↑	AP75↑	mAP↑	AP50↑	AP75↑	mAP↑	AP50↑	AP75↑
Baseline	28.4	48.1	29.5	14.9	25.7	15.2	15.1	36.9	9.9
+MSRB + GSA	28.9	48.8	29.9	16.0	27.9	16.4	15.3	37.7	10.3

The effectiveness of this weighting strategy is evaluated through comparative experiments, and the results are summarized in Table 3. As a baseline, we first consider a simple fusion method based on element-wise addition (+ MSRB + GSA). On the VisDrone dataset, this fusion strategy improves mAP from 28.4% to 28.9%, with AP50 and AP75 improving by 0.7% and 0.4% respectively. Although we can see that the multi-branch fusion is advantageous, it is still not as advantageous as when using a single module (as seen from the ablation studies). Similar trends are also observed on the UAVDT dataset with a rise in mAP from 14.9% to 16.0%, AP50 and AP75 increasing by 2.2% and 1.2% respectively. The improvements on the AU-AIR dataset are less dramatic, going from an mAP of 15.1% to 15.3%, but AP50 and AP75 increase by 0.8% and 0.4%. In general, these results show that although direct feature fusion results in some basic performance improvement, it is unable to selectively suppress redundant interference. Limited by this, CAWM has been proposed that provides more adaptive and efficient feature weighting during the fusion of multi-branch.

#### Ablation study of the ACFM algorithm module

To verify whether the designed adaptive channel ACFM module can effectively achieve accurate detection of small vehicles in complex traffic scenarios, we systematically integrated the ACFM module. Specifically, we first added only the MSRB component to the baseline model, and then successively added the GSA and CAWM components to finally form a complete ACFM module. As shown in Table 4, the experimental results accurately quantified the contribution gain of each component by comprehensively comparing the detection performance of each model variant under different component combinations.

**Table 4:** ACFM ablation experiments on VisDrone, UAVDT, and AU-AIR data (%).

Dataset	Method	MSRB	GSA	CAWM	mAP	AP50	AP75	Params(M)	FLOPS(G)
VisDrone	Baseline				28.4	48.1	29.5	42.52	499.88
		✓			29.1	49.0	30.0	41.08	484.32
			✓		28.8	48.9	30.1	41.08	478.70
				✓	28.9	48.8	29.9	41.08	485.40
	ACFM	✓	✓	✓	**29.2**	**49.1**	**30.2**	**41.08**	**485.42**
UAVDT	Baseline				14.9	25.7	15.2	42.19	499.19
		✓			16.0	27.8	16.9	41.05	483.63
			✓		15.8	27.5	16.5	41.05	478.01
				✓	16.2	27.9	16.4	41.05	484.11
	ACFM	✓	✓	✓	**16.2**	**28.2**	**17.4**	**41.05**	**484.13**
AU-AIR	Baseline				15.1	36.9	9.9	42.51	499.68
		✓			15.6	37.5	10.3	41.07	484.12
			✓		15.5	37.8	10.4	41.07	478.50
				✓	15.3	37.7	10.3	41.07	485.20
	ACFM	✓	✓	✓	**15.6**	**37.9**	**10.4**	**41.07**	**485.22**

**Fig. 5:**
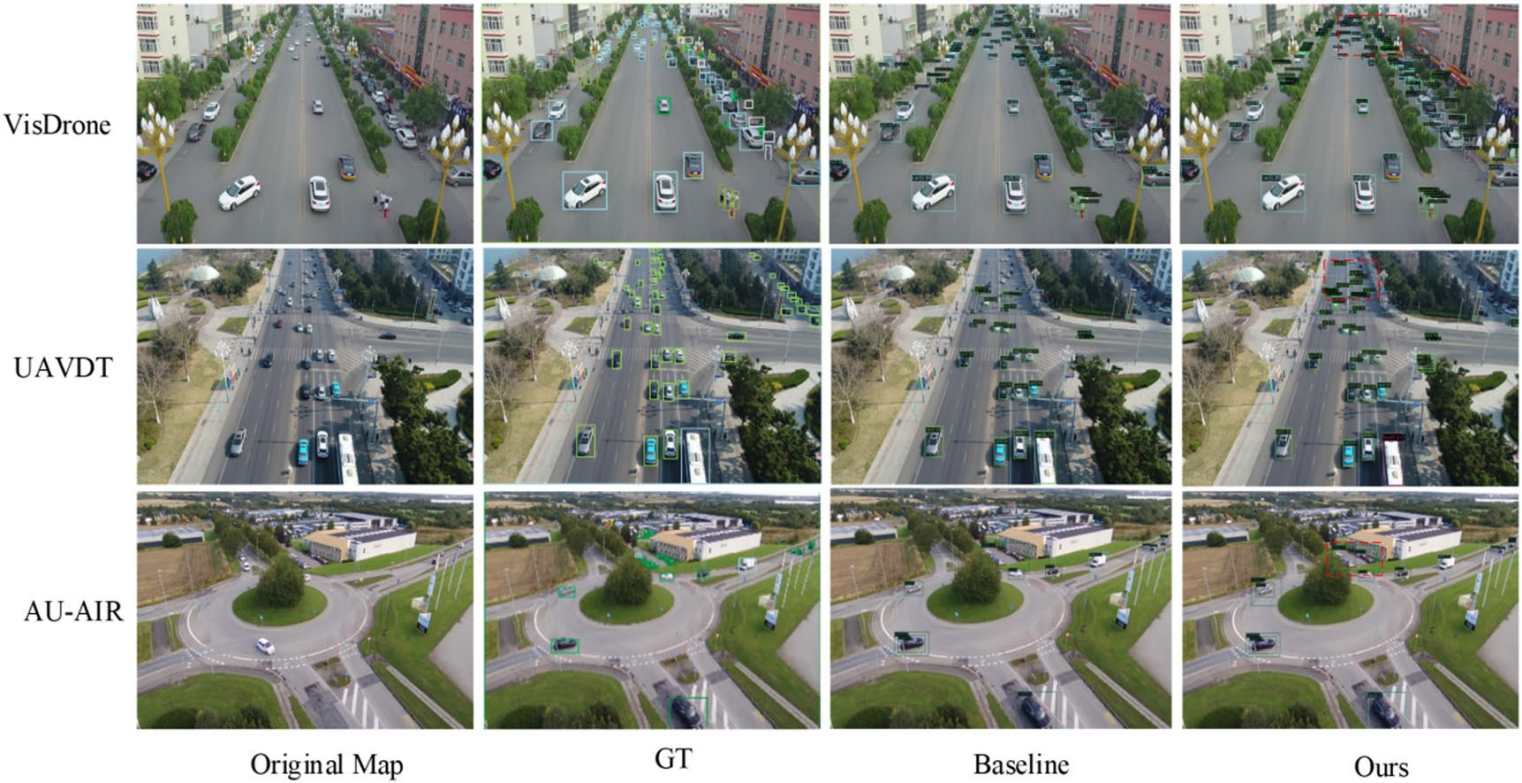
Comparison of detection effects. This figure is composed of images obtained from detection experiments in this study and manually spliced. On the VisDrone, UAVDT, and AU-AIR three datasets, the results of the original images, labeled images, the original algorithm’s detection images, and the detection images of this algorithm are respectively presented for comparison.

Experimental data show that the ACFM full ensemble is the best overall and more efficient. On VisDrone dataset, it raises mAP to 29.2% from 28.4% (AP50 / AP75 + 1.0% / +0.7%), cuts param to 41.08 M and FLOPS to 485.42G. Simple MSRB-GSA fusion underperforms single MSRB, verifying ACFM’s feature conflict resolution. On UAVDT, mAP increases much more obviously (14.9% -> 16.2%), and AP75 is better than simple fusion, indicating more dense-object discrimination. On AU - AIR, it’s mAP up by 0. 5% to 15. 6% and stays lightweight. In general, ACFM can fully blend the advantages of MSRB and GSA and improve local details and suppress interferences and has an excellent balance of accuracy and complexity model. Representative detection results are shown in Fig. 5.

#### Sensitivity analysis of group paradeters in GSA

We perform a sensitivity analysis with respect to the number of groups (G) of the GSA module to test the effect of the channel grouping strategy on feature aggregation. Result shown in Table 5.

**Table 5:** Groups of GSA ablation experiments on VisDrone.

Groups of GSA	mAP(%)	AP50(%)	AP75(%)	Params(M)	FLOPS(G)
8	28.8	48.4	29.4	41.15	488.18
16	29.2	48.7	30.3	41.10	486.34
64	28.7	48.3	29.6	41.08	484.96
32 (ours)	29.2	49.1	30.2	41.08	485.42

From the experiments we can see that the size of group has a big effect in detecting. G = 8 mAP is only 28. 8%, which means inadequate grouping fails to grasp a variety of spatial details. On the other hand, increasing G to 64 leads to a mAP drop to 28. 7%, indicating that excessive fragmentation will interrupt the feature semantic continuity. Moreover, although both G = 16 and G = 32 can reach the peak mAP of 29. 2%, it is the G = 32 configuration that has better robustness and efficiency. Specifically, compared with G = 16, G = 32 can get better AP50 with the cost of small increase in computational cost, which goes from 48.7% to 49.1% while the FLOPS go down a little from 486.34G to 485.42G. Consequently G = 32 is the best compromise, giving a high-precision detection with a limited complexity model.

### Comparisons with state-of-the-the-art

To prove the practical effectiveness of the above method, the mainstream detector and the model on the VisDrone^42^, UAVDT^43^, and AU-AIR^44^datasets were used for comparative experiments. Among the typical detectors GFL^12^, RetinaNet^16^, and FSAF^19^, they were also evaluated on two different backbones: ResNet18 and ResNet50. Output results in Table 6.

**Table 6:** Comparisons with state-of-the-art on VisDrone, UAVDT, and AU-AIR datasets (%).

Methods	Backbone	VisDrone2019			UAVDT			AUAIR		
		mAP↑	AP50↑	AP75↑	mAP↑	AP50↑	AP75↑	mAP↑	AP50↑	AP75↑
GFL	ResNet50	24.3	39.8	25.2	15.6	27.4	16.3	6.4	16.2	4.1
GFL + EFC	ResNet50	30.6	51.1	31.8	16.5	29.0	17.6	13.6	33.8	8.8
GFL+ACFM	ResNet50	31.2	51.8	32.2	17.3	29.6	18.1	14.0	34.5	9.3
RetinaNet	ResNet50	19.4	32.4	20.1	15.7	27.2	17.2	12.0	30.5	7.6
RetinaNet + EFC	ResNet50	24.2	40.7	24.8	15.3	26.4	16.4	16.1	38.6	11.2
RetinaNet+ACFM	ResNet50	24.8	41.3	25.2	15.9	27.8	17.1	16.5	39.7	11.6
FSAF	ResNet18	18.7	34.0	17.7	15.3	27.3	15.7	12.3	31.6	6.9
FSAF + EFC	ResNet18	25.5	47.2	24.7	15.9	28.4	16.4	14.8	36.1	9.1
FSAF+ACFM	ResNet18	26.4	48.3	25.3	16.9	29.8	17.8	15.3	36.6	10.2
FCOS	ResNet50	17.4	30.7	16.8	14.8	27.5	15.1	10.3	25.9	5.8
FCOS + EFC	ResNet50	18.6	32.6	18.7	15.3	27.9	15.5	11.0	28.7	6.4
FCOS+ACFM	ResNet50	19.3	33.2	19.1	16.2	29.0	16.9	13.0	33.2	7.1

The experimental results show that in the three datasets, when the backbone network and the dataset are the same, compared with other algorithms, the proposed ACFM algorithm module has obvious advantages. For Visdrone dataset, backbone network is set to be ResNet50, and detector is set to be GFL. Then, ACFM reaches the final mAP of 31.2% which is higher than FPN by 6.9% and EFC by 0.6%. The RetinaNet dataset, with ACFM reached a MAP of 24.8%, with the FPN being 24.2% and the EFC 19.4%. On FCOS dataset, our method achieved a better mAP by 1.9% and 0.7% than FPN and EFC respectively. ResNet18 as backbone network, ACFM has got mAP improvement up to 7.7% and 0.9% on FSAF dataset. On UAVDT, with ResNet50 backbone and GFL detector, ACFM was 15.6% and 15.8% better than FPN and EFC. On RetinaNet, our method gets 1.7% and 0.8% respectively. For FCOS, ACFM gets higher by 1.4% w.r.t FPN and 0.9% w.r.t EFC. Use ResNet18 as backbone on FSAF dataset, the result shows that ACFM’s mAP is 1.6% and 1.0% higher than traditional FPN and EFC respectively. On the AU-AIR dataset, when using ResNet50 as the backbone network, ACFM reaches 14.0% mAP for GFL detector, it surpasses FPN and EFC with 6.4%, 13.6% by 7.6% and 0.4%. On RetinaNet, ACFM has improved the mAP by 4.5% and 0.4% than before. FCOS is our method increased the mAP by 2.7% and 2.0% respectively. ResNet18 + FSAF as backbone, detector achieved 3.0% and 0.5% more mAP than FPN and EFC respectively. Additionally, local AP50, AP75 has an improvement over our previous method. In general ACFM improves the Universal Detection Performance of a wide range of UAV Datasets, most notably, localization & General Precision have been greatly improved. It also shows the method is better for adapting to scenes and for feature extraction, proving its effective and general.

### Comparison with specialized drone detection models

To test if the proposed approach has the overall best competitiveness we compared ACFM to many more of the latest cutting-edge methods on both the VisDrone and UAVDT benchmarks. Tables 7 and 8: Quantitative Results.

**Table 7:** Comparisons with state-of-the-art on VisDrone.

Method	mAP	AP50	Params(M)	FLOPS(G)
CEASC	28.7	50.7	–	150.18
FBRT-YOLO-S	25.9	42.4	2.9	22.9
YOLC	29.7	52.4	–	–
GFL	24.3	39.8	56.16	552.28
GFL+ACFM(Ours)	31.2	51.8	54.72	537.82

The detection results of ACFM are more correct on the Visdrone dataset than existing methods. It reached a mAP of 31.2% and an AP50 of 51.8%, 1.5% and 2.5% more than YOLC and CEASC. In addition, compared with FBRT-YOLO-S, the mAP of ACFM improves by 5.3%. This means the proposed channel-weighted fusion has its own advantages when it comes to dealing with complicated aerial scenes.

**Table 8:** Comparisons with state-of-the-art on UAVDT.

Method	mAP	AP50	Params(M)	FLOPS(G)
ClusDet	17.3	26.5	–	–
UFPMP-Det	24.6	38.7	–	–
FBRT-YOLO-S	18.4	31.1	2.9	22.9
GFL	15.6	27.4	56.13	551.59
GFL+ACFM(Ours)	17.3	29.6	54.69	537.13

A similar trend can also be seen on the UAVDT dataset. From Table 8 we can see that ACFM gets a mAP of 17.3% and a AP50 of 29.6%. It is also the case that it has an AP50 that is 3.1% higher than ClusDet, so its objects are much more likely to be correctly placed. Though ACFM comes with more computational cost (537.13 GFLOPs), the detection accuracy is equivalent to that of ClusDet, but has higher robustness to the inherent challenges of aerial object detection.

## Conclusions

In this paper, we propose an adaptive channel weighting fusion algorithm module whose core is a MSRB and a GSA. The MSRB improves the localization consistency and detail preservation power of small items in multi-resolution feature maps by means of a downsampling- upsampling course and a residual calibration procedure. GSA reduces background interference using the group processing and sparse gating mechanism, improving the ability to represent edges and high-frequency features of sparse small objects. And then, the channel-level adaptive weighting part flexibly changes what parts get more or less focus based on how complicated the scene is, unlike old ways that always did the same thing for every picture. Experimental results show that ACFM is much better than mainstream methods on VisDrone2019, UAVDT and AU-AIR. On VisDrone2019 and UAVDT, ACFM gets bigger mAP boost compared with baselines, 0.8% more on VisDrone2019, 1.3% more on UAVDT. On even the harder AU - AIR datasets, ACFM only has a small 0.5% lead. Validating the power and capabilities of ACFM module to do multi-scale adaptation and suppression of background. From now on we will continue making refinements of the model’s architecture to be able to have an even stronger model that can tell what objects UAVs have, which gives us even more reason to use this algorithm.

## Data Availability

All datasets used in this study are publicly available. Data supporting the conclusions of this study can be obtained from the following websites: AUAIR: https://aistudio.baidu.com/datasetdetail/186096/1Visdrone2019: https://aistudio.baidu.com/datasetdetail/8923UAVDT: https://aistudio.baidu.com/datasetdetail/261267.

## References

[1] Lin, T. Y. et al. Feature pyramid networks for object detection. In *Proceedings of the IEEE conference on computer vision and pattern recognition*, pp. 2117–2125. (2017).

[2] Tan, M., Pang, R. & Le, Q. V. Efficientdet: Scalable and efficient object detection. In *Proceedings of the IEEE/CVF conference on computer vision and pattern recognition*, pp. 10781–10790. (2020).

[3] Ghiasi, G., Lin, T. Y. & Le, Q. V. Nas-fpn: Learning scalable feature pyramid architecture for object detection. In *Proceedings of the IEEE/CVF conference on computer vision and pattern recognition*, pp. 7036–7045. (2019).

[4] Liu, S., Qi, L., Qin, H., Shi, J. & Jia, J. Path aggregation network for instance segmentation. In *Proceedings of the IEEE conference on computer vision and pattern recognition*, pp. 8759–8768. (2018).

[5] Guo, C., Fan, B., Zhang, Q., Xiang, S. & Pan, C. Augfpn: Improving multi-scale feature learning for object detection. In *Proceedings of the IEEE/CVF conference on computer vision and pattern recognition*, pp. 12595–12604. (2020).

[6] Xiao, Y., Xu, T., Yu, X., Fang, Y. & Li, J. A lightweight fusion strategy with enhanced inter-layer feature correlation for small object detection. *IEEE Trans. Geoscience Remote Sensing* (2024).

[7] Waqas, M. & Naseem, A. Artificial intelligence in sustainable industrial transformation: A comparative study of industry 4.0 and industry 5.0. *FinTech Sustainable Innov.* **1**, A2–A2 (2025).

[8] Momposhi, L. et al. Next-Gen threat hunting: A comparative study of ML models in android ransomware detection. *FinTech Sustainable Innov.* **1**, A3–A3 (2025).

[9] Vasant, M., Ganesan, S. & Ganapath Kumar. and. Enhancing e-commerce security: A hybrid machine learning approach to fraud detection. *FinTech Sustainable Innovation* **1** (2025).

[10] Cai, L., Xu, G. & Niyato, D. ASG: an adaptive Savitzky-Golay method for channel Estimation in Deep-Space communications. *IEEE Trans. Aerosp. Electron. Systems* (2025).

[11] Peng, Z. et al. Deep learning-based CSI feedback for RIS-aided massive MIMO systems with time correlation. *IEEE Wirel. Commun. Letters* **13**.8 (2024): 2060–2064 .

[12] Cai, L. et al. Deep learning based channel Estimation for deep-space communications. *IEEE Trans. Veh. Technology* (2025).

[13] Zhou, T. et al. Transformer network based channel prediction for CSI feedback enhancement in AI-native air interface. *IEEE Trans. Wireless Commun.* **23**, 11154–11167 (2024).

[14] Li, J. et al. Scale-aware fast R-CNN for pedestrian detection. *IEEE Trans. Multimedia*. **20** (4), 985–996 (2017).

[15] Ren, S., He, K., Girshick, R. & Sun, J. Faster r-cnn: Towards real-time object detection with region proposal networks. *Advances in neural information processing systems*, *28*. (2015). 10.1109/TPAMI.2016.2577031 27295650

[16] He, K., Gkioxari, G., Piotr, Dollár & Girshick, R. *Mask r-cnn* (IEEE Transactions on Pattern Analysis & Machine Intelligence, 2017). 10.1109/TPAMI.2018.2844175 29994331

[17] Liu, W. et al. Ssd: Single shot multibox detector. In *European conference on computer vision*, pp. 21–37. Cham: Springer International Publishing. (2016), September.

[18] Liu, C. et al. Yolc: you only look clusters for tiny object detection in aerial images. *IEEE Trans. Intell. Transp. Syst.* **25** (10), 13863–13875 (2024).

[19] Xiao, Y. et al. FBRT-YOLO: Faster and Better for Real-Time Aerial Image Detection. *Proceedings of the AAAI Conference on Artificial Intelligence*. Vol. 39. No. 8. (2025).

[20] Li, X., Wang, W., Wu, L., Chen, S., Hu, X., Li, J., … Yang, J. (2020). Generalized focal loss: Learning qualified and distributed bounding boxes for dense object detection.Advances in neural information processing systems, 33, 21002–21012.

[21] Lin, T. Y., Goyal, P., Girshick, R., He, K. & Dollár, P. Focal loss for dense object detection. In *Proceedings of the IEEE international conference on computer vision*,pp. 2980–2988. (2017).

[22] Tian, Z., Shen, C., Chen, H. & He, T. Fcos: Fully convolutional one-stage object detection. In *Proceedings of the IEEE/CVF international conference on computer vision*,pp. 9627–9636. (2019).

[23] Zhang, H., Wang, Y., Dayoub, F. & Sunderhauf, N. Varifocalnet: An iou-aware dense object detector. In *Proceedings of the IEEE/CVF conference on computer vision and pattern recognition*, pp. 8514–8523. (2021).

[24] Zhu, C., He, Y. & Savvides, M. Feature selective anchor-free module for single-shot object detection. In *Proceedings of the IEEE/CVF conference on computer vision and pattern recognition*, pp. 840–849. (2019).

[25] Li, J. et al. Perceptual generative adversarial networks for small object detection. In *Proceedings of the IEEE conference on computer vision and pattern recognition*, pp. 1222–1230. (2017).

[26] Liu, Z., Gao, G., Sun, L. & Fang, Z. HRDNet: High-resolution detection network for small objects. *arXiv preprint arXiv:2006.07607*. (2020).

[27] Singh, B., Najibi, M. & Davis, L. S. Sniper: Efficient multi-scale training. *Advances in neural information processing systems*, *31*. (2018).

[28] Kisantal, M., Wojna, Z., Murawski, J., Naruniec, J. & Cho, K. Augmentation for small object detection. *arXiv preprint arXiv:1902.07296*. (2019).

[29] Chen, Y. et al. Scale-aware automatic augmentation for object detection. In *Proceedings of the IEEE/CVF conference on computer vision and pattern recognition*, pp. 9563–9572. (2021).

[30] Yang, F., Choi, W. & Lin, Y. Exploit all the layers: Fast and accurate cnn object detector with scale dependent pooling and cascaded rejection classifiers. In *Proceedings of the IEEE conference on computer vision and pattern recognition*, pp. 2129–2137. (2016).

[31] Yang, C., Huang, Z. & Wang, N. QueryDet: Cascaded sparse query for accelerating high-resolution small object detection. In *Proceedings of the IEEE/CVF Conference on computer vision and pattern recognition*, pp. 13668–13677. (2022).

[32] Du, B., Huang, Y., Chen, J. & Huang, D. Adaptive sparse convolutional networks with global context enhancement for faster object detection on drone images. In *Proceedings of the IEEE/CVF conference on computer vision and pattern recognition*, pp. 13435–13444. (2023).

[33] Deng, S. et al. A global-local self-adaptive network for drone-view object detection. *IEEE Trans. Image Process.* **30**, 1556–1569 (2020). 10.1109/TIP.2020.3045636 33360993

[34] Liu, S., Huang, D. & Wang, Y. Learning spatial fusion for single-shot object detection. *arXiv preprint arXiv:1911.09516*. (2019).

[35] Hu, M., Li, Y., Fang, L. & Wang, S. A2-FPN: Attention aggregation-based feature pyramid network for instance segmentation. In *Proceedings of the IEEE/CVF conference on computer vision and pattern recognition*, pp. 15343–15352. (2021).

[36] Hu, J., Shen, L. & Sun, G. Squeeze-and-excitation networks. In *Proceedings of the IEEE conference on computer vision and pattern recognition*, pp. 7132–7141. (2018).

[37] Wang, Q. et al. ECA-Net: Efficient channel attention for deep convolutional neural networks. In *Proceedings of the IEEE/CVF conference on computer vision and pattern recognition*, pp. 11534–11542. (2020).

[38] Qin, Z., Zhang, P., Wu, F. & Li, X. Fcanet: Frequency channel attention networks. In *Proceedings of the IEEE/CVF international conference on computer vision*, pp. 783–792. (2021).

[39] Ouyang, D. et al. Efficient multi-scale attention module with cross-spatial learning. In *ICASSP 2023–2023 IEEE international conference on acoustics, speech and signal processing (ICASSP)* (pp. 1–5). IEEE. (2023), June.

[40] Huang, Z. et al. Ccnet: Criss-cross attention for semantic segmentation. In *Proceedings of the IEEE/CVF international conference on computer vision*, pp. 603–612. (2019).

[41] Woo, S., Park, J., Lee, J. Y. & Kweon, I. S. Cbam: Convolutional block attention module. In *Proceedings of the European conference on computer vision (ECCV)*, pp. 3–19. (2018).

[42] Srinivas, A. et al. Bottleneck transformers for visual recognition. In *Proceedings of the IEEE/CVF conference on computer vision and pattern recognition*, pp. 16519–16529. (2021).

[43] He, K., Zhang, X., Ren, S. & Sun, J. Deep residual learning for image recognition. In *Proceedings of the IEEE conference on computer vision and pattern recognition*, pp. 770–778. (2016).

[44] Carion, N. et al. End-to-end object detection with transformers. In *European conference on computer vision*, pp. 213–229. Cham: Springer International Publishing. (2020), August.

[45] Meng, D., Chen, X., Fan, Z., Zeng, G., Li, H., Yuan, Y., … Wang, J. (2021). Conditional detr for fast training convergence. In Proceedings of the IEEE/CVF international conference on computer vision, pp. 3651–3660.

[46] Wang, Y., Zhang, X., Yang, T. & Sun, J. Anchor detr: Query design for transformer-based detector. In *Proceedings of the AAAI conference on artificial intelligence* (Vol. 36, No. 3, pp. 2567–2575). (2022).

[47] Roh, B., Shin, J., Shin, W. & Kim, S. Sparse detr: Efficient end-to-end object detection with learnable sparsity. *arXiv preprint arXiv:2111.14330*. (2021).

[48] Liu, Z., Lin, Y., Cao, Y., Hu, H., Wei, Y., Zhang, Z., … Guo, B. (2021). Swin transformer:Hierarchical vision transformer using shifted windows. In Proceedings of the IEEE/CVF international conference on computer vision, pp. 10012–10022.

[49] Lee, Y., Kim, J., Willette, J. & Hwang, S. J. Mpvit: Multi-path vision transformer for dense prediction. In *Proceedings of the IEEE/CVF conference on computer vision and pattern recognition*, pp. 7287–7296. (2022).

[50] Zhu, P., Wen, L., Du, D., Bian, X., Ling, H., Hu, Q., … Song, Z. (2018). Visdrone-det2018:The vision meets drone object detection in image challenge results. In Proceedings of the European Conference on Computer Vision (ECCV) Workshops, pp. 437–468.

[51] Du, D., Qi, Y., Yu, H., Yang, Y., Duan, K., Li, G., … Tian, Q. (2018). The unmanned aerial vehicle benchmark: Object detection and tracking. In Proceedings of the European conference on computer vision (ECCV), pp. 370–386.

[52] Bozcan, I. & Kayacan, E. Au-air: A multi-modal unmanned aerial vehicle dataset for low altitude traffic surveillance. In *2020 IEEE International Conference on Robotics and Automation (ICRA)*, pp. 8504–8510. (2020).

